# Application and Measurement Properties of the Talk Test in Cardiopulmonary Patients: A Systematic Review

**DOI:** 10.31083/j.rcm2307225

**Published:** 2022-06-24

**Authors:** Ariany Marques Vieira, Edgar Manoel Martins, Amanda Althoff, Daiana Aparecida Rech, Gustavo dos Santos Ribeiro, Darlan Laurício Matte, Marlus Karsten

**Affiliations:** ^1^Physical Therapy Graduate Program (PPGFT), Santa Catarina State University (UDESC), 88080-350 Florianópolis, SC, Brazil; ^2^Research Group on Cardiovascular Health and Exercise (GEPCardio), Santa Catarina State University (UDESC), 88080-350 Florianópolis, SC, Brazil; ^3^Montreal Behavioural Medicine Centre, CIUSSS du Nord-de-l'Île-de-Montréal, Montreal, QC H4J 1C5, Canada; ^4^Physical Therapy Undergraduate Program, Santa Catarina State University (UDESC), 88080-350 Florianópolis, SC, Brazil; ^5^Physical Therapy Undergraduate Program, University of Vale do Itajaí (UNIVALI), 88302-901 Itajaí, SC, Brazil; ^6^Graduate Program in Rehabilitation Sciences (PPG-CR), Federal University of Health Sciences of Porto Alegre (UFCSPA), 90050-170 Porto Alegre, RS, Brazil

**Keywords:** exercise test, heart diseases, cardiorespiratory evaluation, validity

## Abstract

**Background:**

The talk test (TT) evaluates the exercise intensity by measuring speech comfort level during aerobic exercise. There are several application protocols available to assess individuals with cardiopulmonary diseases. However, the measurement properties of the TT were not systematically reviewed yet.

**Methods:**

A systematic review was developed, registered (CRD420181068930), and reported according to PRISMA Statement. Randomized clinical trials, cross-sectional studies, or series cases were identified through multiple databases and were selected if they presented concomitant speech provocation and an exercise test. Included studies were evaluated based on methodological quality (adapted New Castle-Ottawa Scale), descriptive quality (STROBE Statement), and risk of bias (COSMIN bias risk scale).

**Results:**

Ten studies were included. Seven studies presented moderate to high quality and the majority presented good scores according to the STROBE statement. Four hundred and fourteen subjects performed the TT, the majority being patients with coronary artery disease. The test validity was supported by the included studies. Talk Test reliability was considered satisfactory, although only one study presented an adequate reliability analysis. The studies found a correlation between the last positive stage of the TT with the first ventilatory threshold. Workload, oxygen uptake, and heart rate in the last positive stage of the TT were not different from the same parameters related to the first ventilatory threshold.

**Conclusions:**

The evidence indicates that the TT is suitable as an alternative tool for the assessment and prescription of exercise in individuals with cardiovascular diseases. The stage when the individual is still able to speak comfortably is suggested as the intensity for aerobic exercise prescription. As there is still no well-defined and fully explored TT protocol, caution is required when interpreting the TT results.

## 1. Introduction

Exercise training is recommended for cardiovascular disease (CVD) patients aiming to restore their maximum level of activity and promote their cardiovascular adaptation [1, 2]. Usually, the exercise intensity is prescribed based on parameters from the cardiopulmonary exercise test (CPX), which is recommended on a regular basis for evaluation of adults with chronic diseases [3]. The CPX should be applied whenever there is clinical indication and availability [4]. However, some more accessible tools, at lower cost, have been used for prescription and monitorization of the exercise training such as the Borg Scale [5], and the Talk Test [6]. These alternative tools are important especially when the CPX is not available, potentially providing useful parameters to exercise prescription, facilitating the applicability and dissemination of the aerobic exercise intervention. They can also be useful for aerobic exercise prescription to cardiopulmonary patients in a home-based setting or when social distancing is necessary, as it has been experienced in the COVID-19 pandemic. Besides that, these prescription resources may be applied for the large part of the global population that is sedentary and presents a low level of physical activity.

The Talk Test (TT) is a non-invasive procedure that assesses metabolic stress through speech provocation, indicating the ideal intensity of aerobic exercise [6, 7]. Methodologically, the TT is divided into progressive stages with speech provocation at the end of each stage. The subject is asked to recite a paragraph and to answer the question: “Are you able to speak comfortably?”. There are three answer options: “*Yes*”, for positive stages; “*More or less*”, for equivocal or uncertain stages; and “*No*”, which corresponds to the negative stage, a condition used to finish the test [8, 9]. Another strategy for speech-provoking, which is less commonly used, is counting as a continuous measure of ventilatory stress. The subject is required to count out loud at their normal rhythm, with the number of counts attained in one breath used as a measure [7, 10].

There is a conflict between metabolic and phonetic functions, caused by increased gas exchange and reduced expiratory time during exercise [11]. Studies have observed that the first ventilatory threshold identified during CPX (e.g., the moment when pulmonary ventilation starts to increase disproportionally in relation to oxygen consumption), may be associated with the moment when this conflict is insurmountable. This usually happens during the uncertain or negative stage of the TT [12, 13]. Furthermore, the relationship between exercise intensity and physiological thresholds in the TT seems to be maintained even with different strategies for speech provocation [12].

The application of the TT has been explored since 2002 in different populations, such as athletes [13, 14, 15], men with prostate cancer [16], overweight and obese patients [17], and patients with CVD [18]. It is presently a recommended test in the current guidelines for CVD patients even without a clear definition of the most appropriate protocol [19, 20]. In addition, its applicability to patients with pulmonary disease is still unknown. This increased interest and usefulness of the TT as an evaluation and prescription tool for patients with cardiopulmonary diseases calls for further knowledge about its applicability, protocols, and properties. Therefore, the present study aims to synthesize the application methods and measurement properties of the TT in individuals with cardiopulmonary diseases.

## 2. Materials and Methods

This systematic review was registered in the *Prospective Register of Systematic Reviews* (PROSPERO) under code CRD42018106893 and was reported according to the *Preferred Reporting Items for Systematic Reviews and Meta-Analyses* (PRISMA statement) [21].

### 2.1 Data Sources and Searches

The search was performed in CINAHL, EMBASE, LILACS, Pubmed, and Scopus databases (1995–2022), based on PECO strategy: *Population*, individuals with chronic cardiopulmonary diseases; *Exposure*, TT or similar; *Comparison*, CPX, or another available exercise test; *Outcome*, the application methods of the TT as a primary outcome (such as the ergometer used, the speech provocation method, stages duration and exercise protocol) and the measurement properties as secondary outcomes. Additionally, authors screened the bibliography of the selected manuscripts for full reading and performed a search on Research Gate and Google Scholar. The full search strategy is available in Supplemental Material (**Supplementary Table 1**).

### 2.2 Studies’ Selection 

After performing the literature search, retrieves were organized using the reference manager software Mendeley® (Mendeley Desktop, version 1.19.8, Oxford, UK). After removing duplicate titles, the selection process was performed independently by three blinded researchers. The first stage consisted of titles and abstracts screening. At the end of this step, the independent spreadsheets were joined, and the answers were compared. A senior researcher was consulted to resolve disagreement. Next, the full reading of the selected scientific articles was done following the same process to incorporate the manuscripts that met the inclusion criteria.

Therefore, the scientific articles that met the following criteria were included: samples comprised of individuals with chronic cardiovascular and/or pulmonary diseases, of both genders, aged 18 years or older; manuscripts characterized as randomized clinical trials, cross-sectional studies, or case series, without language restriction, indexed in the databases of interest or retrieved from hand search. Scientific articles that did not present speech provocation, literature reviews, protocols, and study designs were excluded.

### 2.3 Data Extraction and Quality Assessment

Characteristics and main findings of the included studies, as well as the characteristics of the TT application protocols and their measurement properties, were extracted and organized in standardized spreadsheets. Measures of validity (TT stages versus CPX variables), reliability (test-retest or intraclass correlation coefficient), measurement error, and responsiveness were the variables of interest, based on the COSMIN Statement [22].

The methodological quality was analyzed using the adapted New Castle-Ottawa Scale (NOS). This scale consists of seven items, subdivided into three domains which are rated by stars [23, 24]: selection (5-stars), comparability (2-stars), and outcome (3-stars). Studies were scored as low (5 stars or less), moderate (6 or 7 stars), or high-quality (8 or more stars) [24].

The descriptive quality was verified applying the STROBE Statement, which contains 22 items divided into six categories [25, 26]. One point was assigned to each item present in the study. Besides, the COSMIN Risk of Bias Scale [22] was used. This scale is composed of 10 boxes that can be chosen separately according to the evaluation needs and the properties explored by the studies. In this systematic review, for the bias risk analysis, we selected boxes 3 and 8 (validity) and box 6 (reliability). Each box has up to five response options in descending order: very good, adequate, doubtful, inappropriate, and not applicable. The scientific articles’ rating was based on the lowest response for each evaluated box.

## 3. Results

### 3.1 Selected Studies

**Fig. 1.**
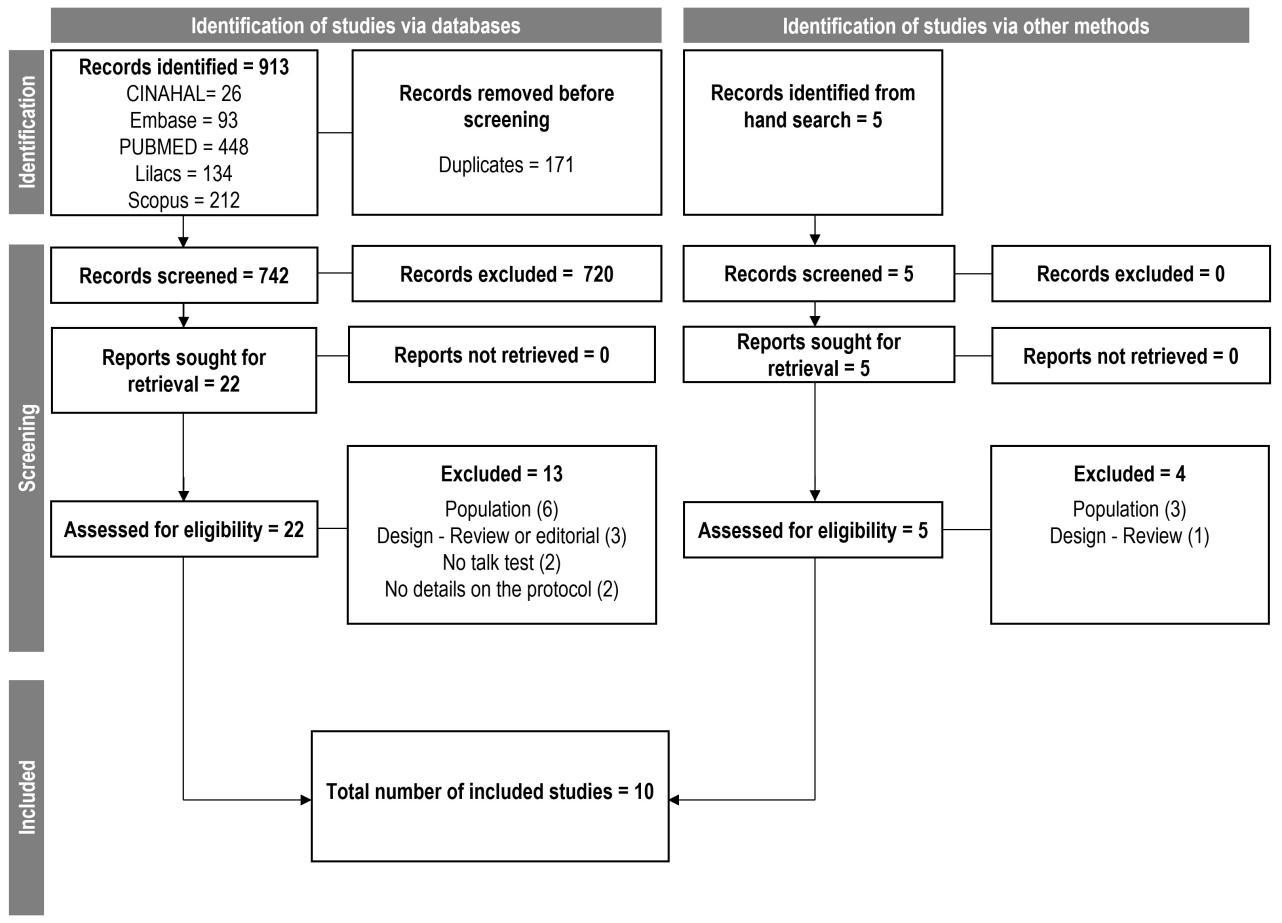
**Flowchart of the screening process and studies selection**.

Fig. 1 shows the process of manuscript selection. The searches retrieved 913 references and five were retrieved from grey literature. Researchers identified 22 references from the main search for a full-text assessment, plus the five references from hand search. The main reason for excluding studies after the full-text assessment was the population (e.g., not cardiopulmonary patients). The study design was also a common reason, as there was no original data on the application of the TT, but a review of the literature for instance. After the full-text assessment, ten studies met the inclusion criteria and were analyzed.

### 3.2 Studies Characteristics

Nine studies were classified as observational and one as a validation study (Table 1, Ref. [18, 27, 28, 29, 30, 31, 32, 33, 34, 35]). Four hundred and twenty-six individuals underwent the TT. From those, the vast majority (more than 99%) were CVD patients, with a predominance of coronary artery disease. Only one study included subjects with chronic pulmonary disease in their sample [32]. The sample size varied from 10 to 85 patients included in each study (mean 42.6 ± 25.18 patients).

**Table 1.** **Characteristics of the included studies**.

Author, Year	Disease	Age*	Sample Size	Country	Design	Main aim	Main results	NOS (★)	STROBE
Brawner, 2006 [27]	CAD	62 ± 9	24	USA	OE	To evaluate the exercise intensity response using the TT during 2 different speech provocation strategies.	A strong correlation between HR in the last TT+ stage of TT-TM and TT-track was found (r = 0.71). The mean HR difference between these methods was 0 ± 16 bpm with an EPI of 1 ± 2 bpm.	High	20/22
								(8)	
Cannon, 2004 [28]	IHD	59 ± 10	19	USA	OE	To examine the relationship between TT and MI threshold in individuals with CVD.	TT+ preceded the ischemic threshold in 84.2% of subjects. Were found moderate correlation between ischemia and TT+ (r = 0.40), TT± (r = 0.39), and TT- (r = 0.32).	Moderate	20/22
								(7)	
Krawcyk, 2017 [29]	Lacunar stroke	67 (44–85)	60	DK	OE	To investigate the viability and reproducibility of TT in individuals with lacunar stroke.	TT reliability was extremely high. For TT-, Bland-Altman analysis confirmed no heteroscedasticity.	High	22/22
								(8)	
Lyon, 2014 [30]	10 RM	66 ± 9	30	USA	OE	To assess the TT responses for providing the appropriate training intensity for individuals in CRP.	TT+ represents 88% ${\mathrm{VT}}_{1}$, PP-1 represents 77% ${\mathrm{VT}}_{1}$ and PP-2 represents 65% ${\mathrm{VT}}_{1}$. However, no maximal exercise test was performed, the authors inferred that the TT± is related to the ${\mathrm{VT}}_{1}$.		18/22
	13 PCI								
	6 AMI								
	5 VS							Low	
	1 ablation 1 AA							(5)	
	1 AAA								
	1 HF								
Nielsen, 2014 [31]	IHD	36 to 82	64	DK	OE	To evaluate the relative reliability and measurement error of incremental cycle ergometer testing with TT for individuals with heart disease.	ICC of 0.90, 0.91, and 0.90 were observed for TT+, TT±, and TT-, respectively. The physiotherapist’s ICCs ranged from 0.81 to 0.88. Excellent relative reliability was observed. The absolute reliability of TT-ICT was lower in 2 stages of the exercise protocol.	Moderate	21/22
								(7)	
Nielsen, 2016 [32]	81 IHD 16 CABG 29 PCI	63 ± 10	85	DK	OE	To investigate the responsiveness of TT-ICT protocol to detect clinically relevant changes over time for individuals participating in CRP.	A 30 W change in this test protocol is suggested as MCID. Significant improvement of TT-ICT was observed in 36% of subjects (surpassed 2 test stages).		20/22
	13 angina								
	8 VS							High	
	4 cardiac arrest							(8)	
	2 COPD								
Petersen, 2014 [33]	18 valve disease	66.5	64	DK	OE	To evaluate the absolute and relative inter-rater reliability of TT in cardiac subjects.	The inter-rater reliability of TT was poor. TT is an insufficient measure to monitor exercise intensity of heart disease patients when 2 or more therapists administer TT.		22/22
	10 HF	(31–86)						Moderate	
	24 CAD with CABG							(7)	
	12 CAD with PCI								
Sorensen, 2020 [34]	8 CABG	65 ± 8.5	20	DK	OE	To evaluate the relationship between the TT and ventilatory threshold measured with gas analysis in a population with cardiac disease.	The workload and ${\mathrm{VO}}_{2}$ values were not different between the stages TT± and TT- but showed a wide range when compared to ${\mathrm{VT}}_{1}$ (r = 0.45 for workload and r = 0.54 for ${\mathrm{VO}}_{2}$, both between TT± and ${\mathrm{VT}}_{1}$). However, the intensity was also not different from the two stages and the ${\mathrm{VT}}_{1}$. HR showed the weakest correlation (r = 0.37) between TT± and ${\mathrm{VT}}_{1}$. For HR, there was a similar intensity between TT± and TT- and the ${\mathrm{VT}}_{1}$ while the intensity at TT- was higher than at ${\mathrm{VT}}_{1}$.		17/22
	6 PCI							Low	
	4 Heart valve surgery							(5)	
	2 HF								
Voelker, 2002 [18]	IHD	63 ± 3	10	USA	OE	Expand the use of TT for stable CVD subjects.	There was a significant difference in ${\mathrm{VO}}_{2}$ and HR between ${\mathrm{VT}}_{1}$and the TT- stage, but not for the TT+ and TT± stages. There was a good correlation between ${\mathrm{VO}}_{2}$ at ${\mathrm{VT}}_{1}$ and the TT+ ($R^{2}$ = 0.53), TT± ($R^{2}$ = 0.51), and TT- ($R^{2}$ = 0.67) stages of TT.	Low	15/22
								(5)	
Zanettini, 2012 [35]	Recent CABG	60 ± 14	50	IT	validation study	To validate TT for exercise prescription in individuals participating in CRP after recent CABG or PCI.	No differences were found between the load assessments of individuals and physical therapists at different stages of TT. Using TT+ to optimize the intensity of aerobic training after recent myocardial revascularization is an effective and safe strategy.	Moderate	21/22
								(7)	

*Average age ± standard deviation or mean (minimum–maximum). CAD, coronary artery disease; USA, United States of America; OE, observational study, TT, talk test; HR, heart rate; TT+, the last positive stage of the talk test; TT-TM, treadmill talk test; TT-track, indoor track talk test; $R^{2}$, determination coefficient; EPI, effort perception index; IHD, ischemic heart disease; MI, myocardial infarction; CVD, cardiovascular disease; TT±, uncertain stage of the talk test; TT-, negative stage of the talk test; DK, Denmark; PCI, percutaneous coronary intervention; AMI, acute myocardial infarction; VS, valve surgery; AA, aortic aneurysm; AAA, abdominal aortic aneurysm; HF, heart failure; ${\mathrm{VT}}_{1}$, first ventilatory threshold; PP-1, penultimate positive stage of talk test; PP-2, antepenult positive stage of talk test; ICC, Intraclass correlation coefficient; TT-ICT, incremental cycle ergometer talk test; COPD, chronic obstructive pulmonary disease; CRP, cardiovascular rehabilitation program; MCID, minimal clinically important difference; CABG, coronary artery bypass grafting; IT, Italy.

Overall, the TT protocol used was a reproduction of the maximal exercise test (Table 2, Ref. [18, 27, 28, 29, 30, 31, 32, 33, 34, 35]). Seven studies applied a single protocol to assess all subjects, without adjusting or individualizing the load increment. The tests were performed on a cycle ergometer (60%) [29, 31, 32, 33, 34, 35], or on a treadmill (20%) [18, 28]. In one study (10%) individuals could choose the ergometer test based on their preference and exercise capacity [30], while another study (10%) applied the TT on both a treadmill and an indoor track [27].

**Table 2.** **Talk Test exercise protocol and quality of the measurement properties**.

Author	Ergometer	Protocol	Duration of stages (min)	Load progression	Evaluation	Metric Properties	Reliability*	Validity*
Brawner, 2006 [27]	Treadmill and indoor track	TT-TM: the same protocol of the maximum test.	2 or 3	TT-TM: fixed	TT-TM: Can you speak comfortably? Yes (+), No (-), or Uncertain (±)	-	-	-
		TT-track: to walk at the fastest pace that still allowed them to speak comfortably.		TT-track: adjusted	TT-track: Questions recorded on a portable device for individuals to answer and guide their exercise speed (no evaluation of different stages).			
Cannon, 2004 [28]	Treadmill	Graduated exercise test: Bruce protocol.	2	Fixed	Can you speak comfortably? Yes (+), No (-), or Uncertain (±)	-	-	-
Krawcyk, 2017 [29]	Cycle	Patients cycled – 2 minutes (15 W) and 60 rpm in 15 W stages.	1	Fixed	Can you speak comfortably? Yes (+), No (-), or Uncertain (±)	TT+ = ICC 0.97 (0.87 to 0.95); SEM = 10.6 W; MDC = 29.4 W	Doubtful	-
						TT- = ICC 0.97 (0.95 to 0.98); SEM = 6.6 W; MDC = 18.3 W		
Lyon, 2014 [30]	Cycle or treadmill	Cycle: ↑10–20 W each stage.	2	Fixed	Can you speak comfortably? Yes (+), No (-), or Uncertain (±)	-	-	-
		Treadmill: modified Balke protocol.						
Nielsen 2014, 2016 [31, 32]	Cycle	Same as the maximum test: Graded cycling test 60 rpm in 15 W stages.	1	Fixed	Can you speak comfortably? Yes (+), No (-), or Uncertain (±)	TT+ = ICC 0.9 (0.84 to 0.94); SEM = 8.9 W; MDC = 24.7 W	Doubtful	-
						TT± = ICC 0.91 (0.83 to 0.94); SEM = 8.8 W; MDC = 24.4 W		
						TT- = ICC 0.90 (0.83 to 0.94); SEM = 9.3 W; MDC = 25.9 W		
Petersen, 2014 [33]	Cycle	Same as the maximum test: submaximal ramp test – 2 minutes unloaded (0 W) and 60 rpm in 15 W stages.	1	Fixed	Assessed if the patients could read aloud without further inspiration. In case they could, the TT was passed, and the exercise continued. If the TT was not passed, it was defined as a negative TT (-).	ICC = 0.85 (0.78 to 0.91)	Proper	-
						SEM = 11W (10 to 14)		
						MDC = 32 W		
Sorensen, 2020 [34]	Cycle	Same as the maximum test: standardized ramp protocols with a 15 W increase in workload each minute.	1	Fixed	Can you speak comfortably?	${\mathrm{VO}}_{2}$ - ${\mathrm{VT}}_{1}$ and TT+ (r = 0.60)	-	Inadequate (SV)
					Yes (+), No (-), or Uncertain (±)	Workload - ${\mathrm{VT}}_{1}$ and TT+ (r = 0.51)		Inadequate (CV)
						HR - ${\mathrm{VT}}_{1}$ and TT+ (r = 0.38)		
						${\mathrm{VO}}_{2}$ - ${\mathrm{VT}}_{1}$ and TT± (r = 0.54)		
						Workload - ${\mathrm{VT}}_{1}$ and TT± (r = 0.45)		
						HR - ${\mathrm{VT}}_{1}$ and TT± (r = 0.37)		
						${\mathrm{VO}}_{2}$ - ${\mathrm{VT}}_{1}$ and TT- (r = 0.58)		
						Workload - ${\mathrm{VT}}_{1}$ and TT- (r = 0.45)		
						HR - ${\mathrm{VT}}_{1}$ and TT- (r = 0.39)		
Voelker, 2002 [18]	Treadmill	Same as the maximum test: increase of 2.5% each stage (modified Balke protocol).	2	Fixed but with initial load adjusted	Can you speak comfortably? Yes (+), No (-), or Uncertain (±)	${\mathrm{VT}}_{1}$ and TT+ (r = 0.71),	-	Inadequate (SV)
						${\mathrm{VT}}_{1}$ and TT± (r = 0.75),		
								Very Good (CV)
						${\mathrm{VT}}_{1}$ and TT- (r = 0.83).		
Zanettini, 2012 [35]	Cycle	Not clear whether the initial load was the same as the maximal test. And the load was increased by 10W each stage.	3	Fixed but with initial load adjusted	Can you speak comfortably? Yes (+), No (-), or Uncertain (±)	Reliability of TT stages evaluated by patients and by physiotherapists, considering workload and: TT+ (R = 0.81), TT± (R = 0.81), and TT- (R = 0.85).	Inadequate	Doubtful (SV)
								Very Good (CV)

* COSMIN risk bias scale. TT-TM, treadmill talk test; TT-track, indoor track talk test; ICC, intraclass correlation coefficient; SEM, standard error of measurement; MDC, minimal detectable change; NR, not reported; TT+, the last positive stage of the talk test; TT±, the first uncertain stage of the talk test; TT-, the negative stage of the talk test; ${\mathrm{VT}}_{1}$, first ventilatory threshold; SV, structural validity; CV, criterion validity; R, correlation coefficient.

The exercise test protocol consisted of one-minute stages [29, 31, 32, 33, 34], two-minute stages [18, 28, 30], and three-minute stages [35]. The study that applied two different protocols, had one with two-minute stages and the other with three-minute stages [27]. The speech provocation was done within the last 10 to 30 seconds of each stage and reciting a standard paragraph was the strategy applied in all studies. Only one study applied an additional challenge method [27].

The speech provocation was based either on paragraphs that were well-known to the individuals, such as a 30-word Danish text passage [29, 31, 32], the “Pledge of Allegiance” (a 31-word paragraph widely known within the US population) [18, 27, 28, 30] and the 19th article on the religious freedom of the Italian Constitution [35]. Two studies did not detail the paragraph, just mentioned that had 30 words [33, 34].

As can be observed in Table 2, most studies used the question, “Can you still speak comfortably?” to evaluate the TT stages, giving three answers as options (Yes, Uncertain, or No). Brawner *et al*. [27] used previously recorded questions as a method of speech provocation in the application of the TT in an indoor track. The individuals listened to the questions through a portable music player and answered these to understand their speech comfort level. Another method applied was evaluating whether the individual could read the paragraph in 10 seconds at a constant pace, without looking breathless. If the individual could not complete the reading requiring further inspiration, the TT was defined as negative and the test was finished [33].

### 3.3 Measurement Properties

The Talk Test validity has been investigated in only four studies [18, 27, 30, 35]. In general, it was identified that the workload, the oxygen uptake (${\mathrm{VO}}_{2}$), or the heart rate in the last positive stage (TT+) was not different from the same parameters related to the first ventilatory threshold (${\mathrm{VT}}_{1}$). When subjects were on TT+ or the first uncertain stage of the TT (TT±) they were on or below ${\mathrm{VT}}_{1,}$ and when participants reached first negative stage (TT-), they were surely above their ${\mathrm{VT}}_{1}$. The respiratory compensation point, also known as second ventilatory threshold (VT2), was not explored.

From these findings, the studies proposed the TT+ stage as a prescription parameter and identified its usefulness in most of the individuals evaluated [18, 27, 35]. Sorensen *et al*. [34] found correlations between the TT stages and ${\mathrm{VT}}_{1}$ from 0.37 to 0.60. Also, it was observed that individuals are unlikely to show electrocardiographic signals of myocardial ischemia at the load corresponding to the time they can still speak comfortably (TT+) [28].

The concurrent validity was analyzed from the correlation between ${\mathrm{VT}}_{1}$ and the stages of TT (Table 2) [18, 35]. The structural validity was classified as *doubtful * [35] and as *inappropriate * [18], while the criterion validity was rated *very good* in both studies (**Supplementary Table 3**). The reliability was evaluated in four studies [29, 31, 33, 35] and the values found were high (intraclass correlation coefficient; ICC >0.8).

Nielsen *et al*. [31] found good reliability for both the individuals and the physiotherapists’ evaluations after an incremental cycle ergometer test associated with TT. Similar results were observed in individuals with lacunar strokes [29], and myocardial revascularization [35]. Although an acceptable ICC value was found, Petersen *et al*. [33] observed low reliability among evaluators when the TT was assessed only by physiotherapists and not by participants. The reliability values were classified as adequate [33] in only one study, two studies presented doubtful [29, 31] reliability, and one was classified as inadequate [35] (**Supplementary Table 3**).

### 3.4 Quality Assessment

According to NOS, the methodological quality was considered high in three studies [27, 29, 32], moderate in four [28, 31, 33, 35], and low in other three studies [18, 30, 34] (Table 1 and **Supplementary Table 2**). The descriptive quality scores ranged from 15 to 22, with 22 being the maximum value achieved (STROBE Statement checklist, Table 1). Study size and funding were the items less described. All studies met the requirements for the categories from item 2 (background) to 9 (bias), and only one study [34] did not complete the four discussion categories.

## 4. Discussion

To our knowledge, this is the first review to systematically synthesize the application methods and the measurement properties of the TT in individuals with chronic cardiopulmonary diseases. The present review included ten studies that showed the feasibility of the TT application and its usefulness in individuals with chronic cardiopulmonary diseases, mainly in coronary artery disease patients. Most of the studies included a small sample size and replicated the maximal exercise test to the TT protocol. The TT+ was related to the intensity of aerobic exercise prescription corresponding to the ${\mathrm{VT}}_{1}$. However, the studies present a divergence between the protocols of the TT application and the findings regarding the validity and reliability of the test.

There are several studies in the literature that support the relationship between the TT stages and ventilatory thresholds [18, 36, 37]. However, this relationship may change depending on the population. When applied to healthy individuals, the TT± stage has been correlated with the ${\mathrm{VT}}_{1}$ [37]. Quinn & Coons found that when exercising at the TT+ level, individuals were within the recommended training zone based on ${\mathrm{\%VO}}_{2}$max, % of maximum heart rate and subjective rating of *perceived exertion * [38].

For both CVD patients and healthy individuals, there are discrepancies in the responses due to the different forms of the TT application. The method of speech provocation, mainly the paragraph length, can influence the response [12]. Schroeder *et al*. [39] reported that long paragraphs may result in lower ${\mathrm{VT}}_{1}$, for instance, but with lower prediction errors. This is aligned with the studies included in this review, which did not show much variation in this respect as most studies used a 30-word paragraph. The duration of each TT stage, when the load is increased and the subject answers the speech provocation, is also a component of the TT protocol that can affect subjects’ response. Stages with a longer duration produce less prediction error. The load reached by the individuals in the points equivalent to the ventilatory thresholds significantly decreases when the stages duration increase [40].

Despite the diversity in the TT protocols and the resulting difficulty in arriving at conclusions, the reliability of the TT was satisfactory [29], both for the assessment by the individual and the physiotherapist [31]. Good results regarding the TT reliability in the context of cardiovascular rehabilitation (CR) were found [41, 42]. However, Petersen *et al*. [33] found poor reliability when the test was administered by more than one evaluator, even with acceptable ICC values.

Nevertheless, when assessed by the COSMIN scale [22], only one study achieved the “adequate” rating for its reliability assessment design [33]. It is noteworthy that the reliability can be increased by standardizing the TT protocols (e.g.,: ergometer, load increase pattern, speak provocation method, stage duration,…), which would reduce the variation of the test parameters. Among the measurement properties, it is noted that the validity and reliability of a test are critical to ensure that the same can be replicated and used to relate to clinical outcomes [43].

Although systematic reviews are not available on the present topic, literature and narrative reviews shed light on the gaps and strengths around the TT. In a narrative review of major contributions to the literature, Foster *et al*. [12] presented the TT applicability for exercise assessment and prescription. The review showed strong evidence regarding the heterogeneity of TT protocols, also presented here in individuals with cardiopulmonary diseases. In agreement with other review articles [6, 44, 42], Foster *et al*. [12] demonstrated the physiological mechanisms involved in the relationship between the TT stages and ventilatory thresholds, reinforcing that TT can allow aerobic exercise practice at optimal intensities when the intensity is adjusted to the highest level of comfortable speech.

The TT was well tolerated by individuals with ischemic heart disease, and its application is safe and effective [35] with minimal measurement errors [31]. Its good clinical applicability is related to its easy administration and low cost [6]. This can potentially impact the CR context, in which the exercise component is very beneficial to patients with CVD [1, 20]. Although there are still barriers to CR implementation, its importance has been well consolidated in the literature, showing a positive impact on several risk factors and on reducing the risk of adverse health events [1, 19, 20]. Among the parameters used for exercise prescription (frequency, intensity, time, type), the intensity seems to be the one that most requires attention so that the benefits of the exercise component of CR may be acquired. The TT can be an alternative tool that has the potential to facilitate exercise prescription and self-monitoring in the recommended target intensity [6, 18, 42]. As highlighted by Saini *et al*. [42], the TT can be useful especially in contexts like India, where low-cost and more accessible tools are necessary to overcome some causes of the CR underutilization.

Investigation of the TT in future research may include establishing standards for the application protocol in individuals with cardiovascular and pulmonary disease, as well as assessing responsiveness after rehabilitation programs. Because of the exploratory nature of this study, future research is warranted. Furthermore, it is necessary to evaluate the tolerance and validity of the test when applied exclusively to individuals with pulmonary diseases, since these individuals may present some peculiarities related to dyspnea resulting from dynamic hyperinflation [45].

The present study has some limitations. Although it was not part of the study planning and purpose, it was not possible to meta-analyze the results due to the lack of similarity in the analysis and the presentation of the findings by the included studies. Besides, the tool used to evaluate the quality of studies may not have been sensitive enough, underestimating the classification of most studies concerning their methodological quality (low and moderate). Although the population included by the studies covered in this review is very representative of cardiovascular rehabilitation patients, the present study could not address the impact of functional limitations and various disease stages, affecting the validity of the TT. Evidence is missing regarding the applicability of the talk test in a more heterogeneous population, or as cited previously, pulmonary disease patients that present different physiological responses to exercise. As mentioned previously, future research is warranted, especially to explore the validation of an individualized talk test protocol.

## 5. Conclusions

Our findings reveal important implications for clinical practice and research. Until now, there is no well-defined and in-depth explored TT protocol. The most common protocol is the graded talk test, replicating the maximal exercise test. This protocol is applied on a cycle ergometer, with one-minute stages and asking the subject to read a standardized paragraph to evaluate the speech comfort level. Because of the heterogeneity in protocols and findings, caution is required when applying and interpreting it. The evidence indicates that the TT is suitable, safe, and feasible for assessment and prescription of exercise in individuals with CVD, showing no case of electrocardiographic evidence of myocardial ischemia. The TT+ and TT± stages can be indicated to prescribe exercise when targeting the ${\mathrm{VT}}_{1}$. The TT is a valid and low-cost tool that has the potential to be applied in clinical practice.

## Supplementary Material

Supplementary material associated with this article can be found, in the online version, at https://doi.org/10.31083/j.rcm2307225.
