# Supplementary material for: Application and Measurement Properties of the Talk Test in Cardiopulmonary Patients: A Systematic Review
Source: Rev Cardiovasc Med. 2022 Jun 24;23(7):225. doi: 10.31083/j.rcm2307225 (PMC11266803; doi:10.31083/j.rcm2307225)
Supplement: Supplementary file 1 [file 2153-8174-23-7-225-s1.pdf]

Supplemental Material 1 – Search strategy

Table 1. Search strategy: terms used for database searches

Databases	Search Strategy
CINAHL	AB (((talk) AND ((exercise) OR test)) OR talk test) AND (((((cardiovascular) OR cardiac) OR lung) AND disease)
LILACS	(tw:(talk AND exercise)) OR (tw:(test)) OR (tw:("talk test")) AND (tw:(cardiovascular OR cardiac OR lung OR pulmonary)) AND (tw:(disease))
SCOPUS	((((talk) AND ((exercise) OR test)) OR "talk test") AND (((((cardiovascular) OR cardiac) OR lung) OR pulmonary) AND disease))
Pubmed	(((talk) AND ((exercise) OR test)) OR "talk test") AND (((((cardiovascular) OR cardiac) OR lung) OR pulmonary) AND disease)
EMBASE	(((talk) AND ((exercise) OR test)) OR "talk test") AND (((((cardiovascular) OR cardiac) OR lung) OR pulmonary) AND disease)

Supplemental Material 2 – *Newcastle-Ottawa Scale* evaluation

Table 2. Adapted Newcastle-Ottawa Scale evaluation

	**Selection**				**Comparability**	**Outcome**		**Total**	**Quality**
**Author year**	**1**	**2**	**3**	**4**	**1**	**1**	**2**		
Brawner 2006	b	a	a	b	a	a	a	8	High
Cannon 2004	b	b	a	b	a	a	a	7	Moderate
Krawcyk 2017	b	a	a	b	a	b	a	8	High
Lyon 2014	b	b	c	b	a	b	b	5	Low
Nielsen 2014	b	a	c	b	a	a	a	7	Moderate
Nielsen 2016	b	a	a	b	a	a	a	8	High
Petersen 2014	b	a	b	b	a	a	a	7	Moderate
Sorensen 2020	c	b	c	b	a	b	a	5	Low
Voelker 2002	c	b	c	b	a	b	a	5	Low
Zanettini 2012	b	b	a	b	a	a	a	7	Moderate

Supplemental Material 3 – COSMIN Bias risk evaluation

Table 3. COSMIN Evaluation

Reliability (COSMIN BOX 6)

Author year	Very good	Adequate	Doubtful	Inadequate	NA	Answer
Krawcyk 2017	2/3/4	1	8		5/6/7	Doubtful
Nielsen 2014	2/3/4	1	8		5/6/7	Doubtful
Petersen 2014	2/3/4/8	1			5/6/7	Adequate
Zanettini 2012	2/3	1	8	4	5/6/7	Inadequate

Structural Validity (COSMIN BOX 3)

Author year	Very good	Adequate	Doubtful	Inadequate	NA	Answer
Sorensen 2020			4	1/3	2	Inadequate
Voelker 2002		1/2	4	3		Inadequate
Zanettini 2012		1/2	3/4			Doubtful

Criterion Validity (COSMIN BOX 8)

Author year	Very good	Adequate	Doubtful	Inadequate	NA	Answer
Sorensen 2020	3			1/2		Inadequate
Voelker 2002	1/3				2	Very good
Zanettini 2012	1/3				2	Very good
